# Effect of ESC (electronic stability control) on tree and pole impacts with focus on rear impacts

**DOI:** 10.1016/j.heliyon.2024.e37591

**Published:** 2024-09-07

**Authors:** David C. Viano, Chantal S. Parenteau, Eric R. Teoh

**Affiliations:** a ProBiomechanics LLC, 265 Warrington Road, Bloomfield Hills, MI, 48304, USA; b Design Research and Engineering, 46475 Desoto Ct, Novi, MI, 48377, USA; c Insurance Institute for Highway Safety, 4121 Wilson Blvd, 6th Floor, Arlington, VA, 22203, USA

**Keywords:** ESC, Off-road impacts, Loss of control, Crash avoidance technologies, Injury prevention

## Abstract

**Objective:**

The effect of ESC (Electronic Stability Control) was investigated for the rate of crash exposure, serious injury and fatality in pole and tree impacts. Field data was analyzed by crash type (front, side, rear and rollover) and model year (MY) before, during and after the implementation of ESC.

**Methods:**

The number of pole and tree impacts was determined for four groups of vehicle model years (MY): 1981–1989 MY and 1990–2002 MY before the introduction of ESC, 2003–2009 during the phase-in of ESC and 2010–2020 MY after essentially all vehicles were equipped with ESC. Collisions were grouped by front, side, rear and rollover. Three databases were analyzed: 1990–2020 FARS, 1990–2015 NASS-CDS and 2017–2020 CISS. Vehicle registration was obtained from IHS Markit to determine the rate of pole and tree impacts per 100,000 registered vehicles. The same vehicle selection criteria was used for vehicle registration and crash data.

**Results:**

Fatalities dropped 65.2 % (95 % CI, 63.0–67.4 %), z = 43.7, p < 0.001 into poles and 60.3 % (95 % CI, 59.0–61.5 %), z = 72.4, p < 0.001 into trees in vehicles equipped with ESC comparing 1990–2002 MY to 2010–2020 MY vehicles. Seriously injured occupants in crashes with poles dropped 75.9 % (95 % CI, 75.0–76.9 %), z = 116, p < 0.001 between 1990 and 2002 MY and 2010–2020 MY vehicles. There was a 65.2 % (95 % CI, 64.4–65.9 %), z = 141, p < 0.001 reduction in tree impacts. The crash exposure to pole impacts dropped 36.0 % (95 % CI, 35.8–36.3 %), z = 252, p < 0.001 from 80.77/100,000 registered vehicles in 1990–2002 MY vehicles to 51.69/100,000 in 2010–2020 MY vehicles. There was a 61.0 % (95 % CI, 60.8–61.2 %), z = 434, p < 0.001 reduction in tree impacts. For rear impacts, fatalities dropped 82.9 % (95 % CI, 71.3–94.4 %), z = 9.37, p < 0.001 into poles and 74.8 % (95 % CI, 67.8–81.9 %), z = 14.8, p < 0.001 into trees. Serious-injury in rear impacts with poles and trees were essentially eliminated in 2010–2020 MY vehicle crashes. There were significant drops in fatalities in side and frontals impacts and rollovers in vehicles equipped with ESC.

**Conclusion:**

ESC helps the driver maintain vehicle heading and significantly reduced the rate of serious injury and fatality in off-road impacts with poles and trees. The benefits of ESC may not be realized with impairments when the driver does not appropriately steer the vehicle.

## 1 Introduction

### 1.1 Effectiveness of ESC (electronic stability control)

Tingvall [1] and Aga [2] published the first field effectiveness studies of ESC at the 18th ESV Conference. They showed very-positive field effectiveness of ESC in avoiding serious-injury in single vehicle and head-on crashes. The initial systems only involved control of engine torque, but they soon evolved to active wheel braking. Tingvall's ESV study was subjected to peer review and published [3].

Farmer [4] found that ESC in passenger vehicles reduced single-vehicle crash risks by 41 % and single-vehicle fatal crash risks by 56 % in the US. Within a month of the Farmer publication, the US domestic car manufacturers announced plans to phase-in ESC as standard equipment on sport utility vehicles. Dang [5] found that single vehicle crashes were reduced by 35 % in passenger cars and 67 % in SUVs in the US. Fatal single vehicle crashes are reduced by 30 % in cars and 63 % in SUVs. The reduction in crashes was statistically significant (χ = 6.64, p < 0.01). The reduction in fatal single-vehicle crashes were also statistically significant (p < 0.01 in SUVs and p < 0.05 in passenger cars). ESC was found to be 1.9 to 2.1 times more effective in SUVs than in passenger cars involved in single-vehicle crashes.

Farmer [6] updated the effectiveness of ESC based on more years of field data. He found that ESC reduced 7 % of all crashes and 41 % single-vehicle crashes. The effects were significantly higher for SUVs than for cars. ESC reduced single-vehicle crash risks by 49 % for SUVs and 33 % for cars. ESC reduced single-vehicle fatal crash risks by 56 % based on all fatal crashes in the United States over four years. The effectiveness estimates were 59 % for SUVs and 53 % for cars, although the difference was not statistically significant. Fatal crash risks were reduced 32%–37 % for SUVs and 25 % for cars in multi-vehicle crashes.

There have been numerous follow-on studies on ESC effectiveness, including meta analyses and reviews [[7], [8], [9]]. These summaries found significant effectiveness with ESC preventing rollovers, loss-of-control and single-vehicle crashes, consistent with the findings of others [[10], [11], [12], [13], [14], [15], [16], [17], [18], [19], [20], [21], [22], [23]]. Riexinger and Gable [24] found ESC effective in preventing run-off-road crashes. Several studies found ESC effective in preventing rollover crashes [25,26]. Others have found effectiveness with more subtle effects with studded tires [27] and rumble strips on the roadway [28]. There have been several general studies on the effectiveness of advanced technologies on motor vehicles, including ESC [[29], [30], [31]]. Simulator studies have complemented the field study effectiveness [32,33]. Vedeby et al. [34] studied the perception of drivers to ESC looking for risk-compensation effects. Rudin-Brown [35] studies ESC effects on driving.

### 1.2 FMVSS 126 electronic stability control

In 2005, NHTSA was involved in various ESC evaluation programs. NHTSA published a NPRM in September 2006 proposing a new standard, FMVSS 126 for electronic stability control systems. The final rule came out in 2007 [36]. NHTSA [37] carried out a regulatory impact analysis on ESC. They estimated that ESC was 13 % effective for passenger cars involved in police reported multi-vehicle crashes. ESC was found most effective in LTVs involved in fatal rollover crashes. They estimated that 10 % of vehicles were equipped with ESC in 2003 and about 29 % in 2006.

FMVSS 126 had a phased-in schedule starting with 55 % of 2009 model year vehicles. By 2011, all new 2012 MY vehicles had to comply. The phase in was: 1) 55 % of vehicles manufactured on or after September 1, 2008 and before September 1, 2009, 2) 75 % of vehicles manufactured on or after September 1, 2009 and before September 1, 2010, 3) 95 % of vehicles manufactured on or after September 1, 2010 and before September 1, 2011 and 4) 100 % of vehicles manufactured on or after September 1, 2011. Most vehicle manufactures had already phase-in ESC ahead of the FMVSS 126 requirements. The NHTSA schedule was “after the fact” with regard to the implementation of ESC in vehicles. By 2009 MY essentially all light vehicles were equipped with ESC.

### 1.3 Narrow object impacts

arrow object impacts usually involve loss of vehicle control, yaw off-road and impact into fixed objects, such as trees, poles and embankments. They are uncommon but can lead to serious injury and death. Holdridge et al. [38] found that tree and pole impacts were associated with an increased probability of fatal injury. IIHS [39] summarized the 2020 Traffic Safety Fact and found that about 20 % of motor vehicle fatalities were with fixed objects [40,41]. 46 % were impacts with trees and 11 % were with utility poles. Occupant fatalities from tree and pole impacts were often associated with poor lighting, alcohol use and young male drivers.

Lund et al. [42] analyzed FARS and reported that frontal crashes accounted for about half of all fatalities. 20 % were impacts with a narrow object. Review of frontal pole impact tests indicated that the pole location relative to the vehicle centerline influenced vehicle deformation and occupant injury [43]. IIHS started the shallow offset frontal crash test to evaluate crashworthiness in narrow impacts [44]. Side impacts with trees or poles are a serious crash type due to limited crush space and proximity to the occupant. Pintar et al. [45] analyzed narrow object side impacts using CIREN data and observed that serious head and chest injuries were most common, when the impact was centered mid-wheelbase. They reported that intrusion, impact direction and interaction with the fixed object were factors in the occupant injury. Side pole tests are part of FMVSS 214. The tests are useful, but many fatal and serious injury impacts into poles and trees involve very high speeds.

Rollovers have injury risks that increase with the number of rolls, except when the vehicle rolls ¼-½ turns and slides into a fixed object, such as a tree, roadside structure or embankment [46]. In these cases, the impact with the fixed object can be the most harmful event causing injury, not the ¼-½ roll. When a significant impact to the vehicle occurs in a rollover, it can complicate the interpretation of injury mechanisms in rollover accidents with respect to the number of quarter rolls [47].

### 1.4 Current study

ESC is known to help drivers maintain vehicle heading on the road reducing off-road excursions. Since poles and trees are off-road objects, ESC may be beneficial at preventing fixed object impacts. The current study analyzes the effectiveness of ESC in preventing serious-to-fatal impacts with trees and poles. Three national databases were sorted by vehicle model year (MY) to include vehicles without ESC, during the phase-in and vehicles equipped with ESC. While ESC is available on new vehicles, the evaluation of its effectiveness is part of the scientific method with new technologies. In this case, the effectiveness of ESC in preventing off-road impacts was determined. The study evaluated if ESC was effective in preventing pole and tree impacts.

## 2 Methods

### 2.1 Crash data sources

Three crash databases were analyzed FARS, NASS-CDS and CISS (www.nhtsa.gov).

FARS (Fatality Analysis Reporting System) cases from the 1990–2020 CY were analyzed for pole and tree impacts. FARS includes fatal motor vehicle crashes on a trafficway open to the public that results in the death of a motorist or a non-motorist within 30 days of the crash [48]. The data has been collected since 1975 and is derived from a census of fatal traffic crashes in the 50 States, the District of Columbia, and Puerto Rico. The FARS database contains 143 different coded elements characterizing the crash, vehicles and people involved. The data comes from state-police accident reports, death certificates, state coroners and medical examiners, state driver and vehicle registration records and emergency medical services records.

NASS-CDS (National Automotive Sampling System-Crashworthiness Data System) cases from 1990 to 2015 CY were analyzed for pole and tree impacts. NASS-CDS is a stratified multiphase, unequal selection probability sample of motor vehicle crashes that are prospectively selected for in-depth investigation by trained teams [49]. NASS-CDS samples minor, serious and fatal crashes involving passenger cars, pickup trucks, vans, large trucks, motorcycles and pedestrian crashes. The vehicles are generally towed from the scene. Data has been collected since 1979 with different acronym names. There are 24 field research teams that investigate about 5000 crashes a year. The teams obtain data and photographs from the crash sites, vehicles and occupants. They interview crash victims and reviewing medical records to records the severity of injuries. The data for 2009–2015 CY are representative of MY 2000+ vehicles, because NASS-CDS excluded vehicles 10 MY and older from investigation starting with the 2009 CY.

CISS (Crash Investigation Sampling System) cases from 2017 to 2020 CY were analyzed for pole and tree impacts. CISS revised and followed NASS-CDS with publicly available data starting in 2017 [50]. It is a nationally, representative sample of fatal and nonfatal passenger vehicle crashes. By 2018, it collected crash data from thirty-two teams that investigate the scene, vehicle damage and injury severities and sources. NASS-CDS and CISS data were merged to estimate the national incidence of tree and pole impacts by crash type, injury severity using inflation factors provided by National Highway Traffic Safety Administration (NHTSA). Only towed vehicles were included in this study. A similar analysis was conducted with FARS.

Weighting the NASS-CDS and CISS samples involved renaming a few variables in the NASS-CDS and CISS files. The NASS-CDS data on the vehicle, crash, occupant and injury was merged, then the CISS data on the vehicle, crash, occupant and injury was merged and finally the NASS-CDS and CISS data were combined. CASEWGT (case weight) was the final weight estimation for the data. The variance was estimated using the Taylor series method [50].

### 2.2 Calendar year (CY) groups

The field data is reported in 4 CY groups according to the availability of ESC (Electronic Stability Control) on the vehicle: 1981–1989 MY and 1990–2002 before the introduction of ESC, 2003–2009 during the phase-in of ESC and 2010–2020 MY after essentially all vehicles were equipped with ESC. The groups provide data on collisions with trees and poles before and after vehicles were equipped with ESC. The 2020 CY cases in CISS and FARS include some 2021 MY vehicles sold late in the 2020 CY. The crash and registration data includes some 2021 MY vehicles. The MY designation used is 2010–2020 but note that the 2020 CY data includes some 2021 MY vehicles.

### 2.3 Crashes

The crash type for the struck vehicle was defined using the initial impact direction in FARS (IMPACT 1) and area of most damage in NASS-CDS and CISS:

Front, excluded rollovers and was defined as.

• GAD1 in (F) in NASS-CDS
• CDCPLANE in (F) and rank = 1 in CISS
• IMPACT1 in (11,12,1) in FARS

Side, excluded rollovers and was defined as.

• GAD1 in (L,R) in NASS-CDS
• CDCPLANE in (L,R) and rank = 1 in CISS
• IMPACT1 in (2,3,4,8,9,10) in FARS

Rear, excluded rollovers and was defined as.

• GAD1 in (B) in NASS-CDS
• CDCPLANE in (B,C,D) and rank = 1 in CISS
• IMPACT1 in (5,6,7) in FARS

Rollover, was defined as ROLLOVER/ROLLTURN >0.

### 2.4 Light vehicles

Only light vehicles were included. Passenger cars, SUVs, minivans and pickup trucks were included in the analysis (coded as BODY TYPE/BODY_TYP values 1–8, 14–17, 20, and 30–39). Only towed vehicles were included in the NASS-CDS and CISS samples.

### 2.5 Tree/pole impacts

The source of impact to the struck vehicle was identified using the most harmful event variable (M_HARM) in FARS and object contacted (OBJCONT) in NASS-CDS and CISS.

• Tree/bush (OBJCONT = 41,42) or M_HARM in (41,42)
• Pole (OBJCONT = 45,50,51,52,53) or M_HARM in (30,31)

### 2.6 IHS Markit (R.L. Polk) vehicle registrations

Vehicle registration data for light vehicles was obtained from IHS Markit (https://ihsmarkit.com/products/auto-market-statistics-vio-vin.html). The data is a compilation of all passenger vehicles that have been registered according to State requirements. In 2013, R.L. Polk and Co. was acquired by IHS Markit automotive solutions. For the FARS analysis, all vehicle MY registrations were used for the same selection of light vehicles used in the crash analyses. For the NASS-CDS analyses, the vehicle MY registration was limited to less than 10 years old matching the selection criteria of NASS-CDS starting in the 2009 CY. The same vehicle MY selection criteria was used in the crash and registration data.

### 2.7 Occupant exposure and injury in NASS-CDS and CISS crashes

The number of occupants exposed to crashes with poles and trees was determined as all occupants with known injury status (MAIS 0+F). The number of occupants with serious injury in crashes with poles and trees was determined as the number of occupants with (MAIS 3+F).

### 2.8 Injury risks in NASS-CDS and CISS

The risk for serious injury was determined by dividing the number of occupants with serious injury or greater (MAIS 3+F) by the number of occupants with known injury status (MAIS 0+F) for the different direction of impact with trees and poles. All calculations were based on the weighted sample. Risks are reported with ± one standard error. Cases with an inflation ratio equal to 0 or with a negative inflation ratio were excluded from the analysis.

### 2.9 Exposure and injury rates

The rate of exposure and serious injury in tree and pole impacts was determined by dividing the number of exposed and seriously injured (MAIS 3+F) occupants in NASS-CDS and CISS by the number of registered vehicles from IHS Markit (R.L. Polk). For CY 2009 and above, registered vehicles with 10 MY and older were excluded.

### 2.10 Fatality rate

The fatality rate was determined by dividing the number of fatalities from FARS by the number of registered vehicles from IHS Markit (R.L. Polk).

### 2.11 Statistics

The significance of the differences in rate of exposure, serious injury and fatality was determined using the z-test of proportions with significant difference if p < 0.01 [51]. The percent change in rate included the 95th confidence interval (CI) in brackets.

### 2.12 Individual NASS-CDS and CISS case review of serious injury crashes with trees and poles

Since pole and tree impacts are a common source of serious and fatal injury after high-speed road departure, spin-out and rear-leading skid, individual NASS-CDS and CISS cases were reviewed to see the type of real-world rear impacts ESC helps avoid. All serious injury (MAIS 3+F) cases with a tree or pole impact to the rear of the struck vehicle were downloaded from the 2004–2015 NASS-CDS and 2017–2020 CISS electronic case databases as part of the earlier study [52]. The cases were not discussed in the earlier study. Each of the cases was reviewed and photographs analyzed to gain an understanding of the crash circumstances and factors involved in the serious injury (MAIS 3+F).

### 2.13 Case summary of fatal impacts with poles and trees in FARS

The fatal impacts with trees and poles in the 2010–2020 FARS were summarized for the driver, environment and other factors in the impact. This provided some information on 115 vehicles and 129 occupants the impacts into trees and poles in vehicles equipped with ESC.

## 3 Results

Table 1 summarizes the pole and tree impacts to the front, side and rear of the vehicle and in rollovers in the 1990–2015 NASS-CDS and 2017–2020 CISS. The first group shows the exposed occupants (MAIS 0+F) in pole and tree impacts shown separately. The second group has the number of occupants with serious injury (MAIS 3+F). Each group includes four MY subgroups of vehicle MY without ESC, a mix during the phase-in of ESC and vehicles with ESC. Overall, impacts into trees and poles were less frequent in the 2010–2020 MY vehicles. There were no serious injury crashes in 2010–2020 MY vehicles in NASS-CDS or CISS involving a rear impact into a tree or pole.

**Table 1:** Pole and Tree Impacts by location on the struck vehicle and Injury outcome from 1990 to 2015 NASS-CDS and 2017–2020 CISS.

**1990–2015 NASS-CDS** **& 2017–2020 CISS**	**Weighted Count of Occupants**							**ESC**
	**Impact Location**							
	**Front**	**Side**	**Rear**	**Rollover**	**Other**	**Unknown**	**Total**	
**Model years**	**Pole Impact Exposure (MAIS 0+F)**							
1981–1989	5,11,792	1,49,624	8569	39,760	1721	56,314	7,67,780	no
1990–2002	10,11,703	4,54,923	24,410	89,671	847	44,863	16,26,415	no
2003–2009	3,55,537	71,291	3680	88,197	727	20,627	5,40,060	mix
2010–2020	2,29,931	25,279	222	57,446	0	75,890	3,88,768	yes
Total	21,08,963	7,01,117	36,881	2,75,074	3295	1,97,695	33,23,024	
**Model years**	**Tree Impacts Exposure (MAIS 0+F)**							
1981–1989	5,22,663	1,78,286	4271	62,953	6308	53,267	8,27,749	no
1990–2002	8,55,918	3,74,634	53,649	1,67,239	16,499	87,089	15,55,027	no
2003–2009	2,78,462	1,56,910	5354	69,362	4918	14,437	5,29,443	mix
2010–2020	1,44,394	25,751	0	23,883	166	32,373	2,26,567	yes
Total	18,01,437	7,35,581	63,275	3,23,436	27,891	1,87,166	31,38,786	
**Model years**	**Pole Impact Serious Injury (MAIS 3+F)**							
1981–1989	37,581	10,133	166	3609	10	578	52,078	no
1990–2002	41,368	17,707	633	10,087	356	689	70,841	no
2003–2009	12,763	5109	361	2823	8	2110	23,174	mix
2010–2020	1781	151	0	1172	0	3266	6371	yes
Total	93,492	33,101	1161	17,691	375	6643	1,52,464	
**Model years**	**Tree Impact Serious Injury (MAIS 3+F)**							
1981–1989	40,339	19,991	761	12,382	1682	2201	77,356	no
1990–2002	70,430	36,314	1495	28,805	1209	2906	1,41,158	no
2003–2009	18,992	7473	407	6250	512	226	33,860	mix
2010–2020	13,462	1209	0	1831	0	1863	18,365	yes
Total	1,43,223	64,987	2664	49,268	3403	7196	2,70,740	

Table 2 summarizes the fatalities in pole and tree impacts to the front, side and rear of the vehicle and in rollovers in the 1990–2020 FARS. The number of rear impacts into trees and poles is smaller in the 2010–2020 MY vehicles, but they still occur.

**Table 2:** Pole and Tree Fatal Impacts by location on the struck vehicle from 1990 to 2020 FARS.

	**Count of Fatalities**						**ESC**
**1990**–**2020 FARS**	**Impact Direction**						
	**Front**	**Side**	**Rear**	**Rollover**	**Oth/Unk**	**Total**	
**Model years**	**Pole Impact Fatalities**						
1981–1989	4111	2471	200	1268	393	8443	no
1990–2002	6948	4877	454	3256	1234	16,769	no
2003–2009	2104	1062	100	1158	657	5081	mix
2010–2020	964	164	23	319	256	1726	yes
Total	14,127	8574	777	6001	2540	32,019	
**Model years**	**Tree Impact Fatalities**						
1981–1989	12,685	5061	484	3298	780	22,308	no
1990–2002	27,901	12,539	1425	9694	3009	54,568	no
2003–2009	9535	3675	498	3565	1862	19,135	mix
2010–2020	3964	631	106	1023	687	6411	yes
Total	54,085	21,906	2513	17,580	6338	102,422	

Table 3 shows the number of registered vehicles from IHS Markit that match the selection criteria for the exposure, serious injury and fatality data in Table 1, Table 2 This provides a measure of exposed vehicles in the field. The same limitations on the vehicle age in NASS-CDS was used to determine the number of registered vehicles.

**Table 3:** Registered vehicles from IHS Markit matching the selection criteria for injury and fatal crashes into poles and trees.

**1990–2015 NASS-CDS**	**1990**–**2015**^a^**& 2017–2020 IHS Markit**^b^	**1990**–**2020 FARS**	**1990**–**2020**	**ESC**
**& 2017–2020 CISS**			**IHS Markit**	
**Model years**	**Registered Veh. Years**	**Model years**	**Registered Veh. Years**	
1981–1989	1,143,383,365	1981–1989	1,231,309,952	no
1990–2002	2,013,572,623	1990–2002	2,889,811,700	no
2003–2009	913,229,053	2003–2009	1,372,915,195	mix
2010–2020	752,186,782	2010–2020	854,292,435	yes
Total	4,822,371,823	Total	6,577,759,231	

a For 2010+ NASS-CDS, registrations are limited to vehicles <10 years old per change in NASS-CDS slelection criteria.

b Formerly R.L. Polk and Company.

Table 4 shows the rate of pole and tree impacts by location on the struck vehicle based on the 1990–2015 NASS-CDS, 2017–2020 CISS and 1990–2015 and 2017–2020 IHS Markit data. The rate of exposed and seriously injured occupants was lower in newer vehicles equipped with ESC. The drops are statistically significant in a comparison of 1990–2003 MY vehicles to the 2010–2020 MY vehicle crash rates.

**Table 4:** Rate of Pole and Tree Impacts by location on the struck vehicle and Injury outcome from 1990 to 2015 NASS-CDS, 2017–2020 CISS and 1990–2015 and 2017–2020 IHS Markit.

**CDS 1990–2015 & CISS 2017**–**2020**							**ESC**
**1990**–**2015 NASS-CDS**	**Rate per 100,000 Registered Vehicles**						
**& 2017–2020 CISS**	**Impact Location**						
**1990**–**2015 & 2017–2020 IHS Markit**	**Front**	**Side**	**Rear**	**Rollover**	**Other**	**Total**	
**Model years**	**Pole Impact Occupant Exposure (MAIS 0+F)**						
1981–1989	44.76	13.09	0.75	3.48	0.150	67.15	no
1990–2002	50.24	22.59	1.21	4.45	0.042	80.77	no
2003–2009	38.93	7.81	0.40	9.66	0.080	59.14	mix
2010–2020	30.57	3.36	0.03	7.64	0.000	51.69	yes
Total	43.73	14.54	0.76	5.70	0.068	68.91	
**Model years**	**Tree Impact Occupant Exposure (MAIS 0+F)**						
1981–1989	45.71	15.59	0.37	5.51	0.552	72.39	no
1990–2002	42.51	18.61	2.66	8.31	0.819	77.23	no
2003–2009	30.49	17.18	0.59	7.60	0.539	57.97	mix
2010–2020	19.20	3.42	0.00	3.18	0.022	30.12	yes
Total	37.36	15.25	1.31	6.71	0.578	65.09	
**Model years**	**Pole Impact Serious Injury (MAIS 3+F)**						
1981–1989	3.29	0.886	0.0146	0.316	0.001	4.55	no
1990–2002	2.05	0.879	0.0315	0.501	0.018	3.52	no
2003–2009	1.40	0.559	0.0395	0.309	0.001	2.54	mix
2010–2020	0.24	0.020	0.0000	0.156	0.000	0.85	yes
Total	1.94	0.686	0.0241	0.367	0.008	3.16	
**Model years**	**Tree Impact Serious Injury (MAIS 3+F)**						
1981–1989	3.53	1.748	0.0666	1.083	0.147	6.77	no
1990–2002	3.50	1.803	0.0742	1.431	0.060	7.01	no
2003–2009	2.08	0.818	0.0446	0.684	0.056	3.71	mix
2010–2020	1.79	0.161	0.0000	0.243	0.000	2.44	yes
Total	2.97	1.348	0.0552	1.022	0.071	5.61	

**Fig. 1:**
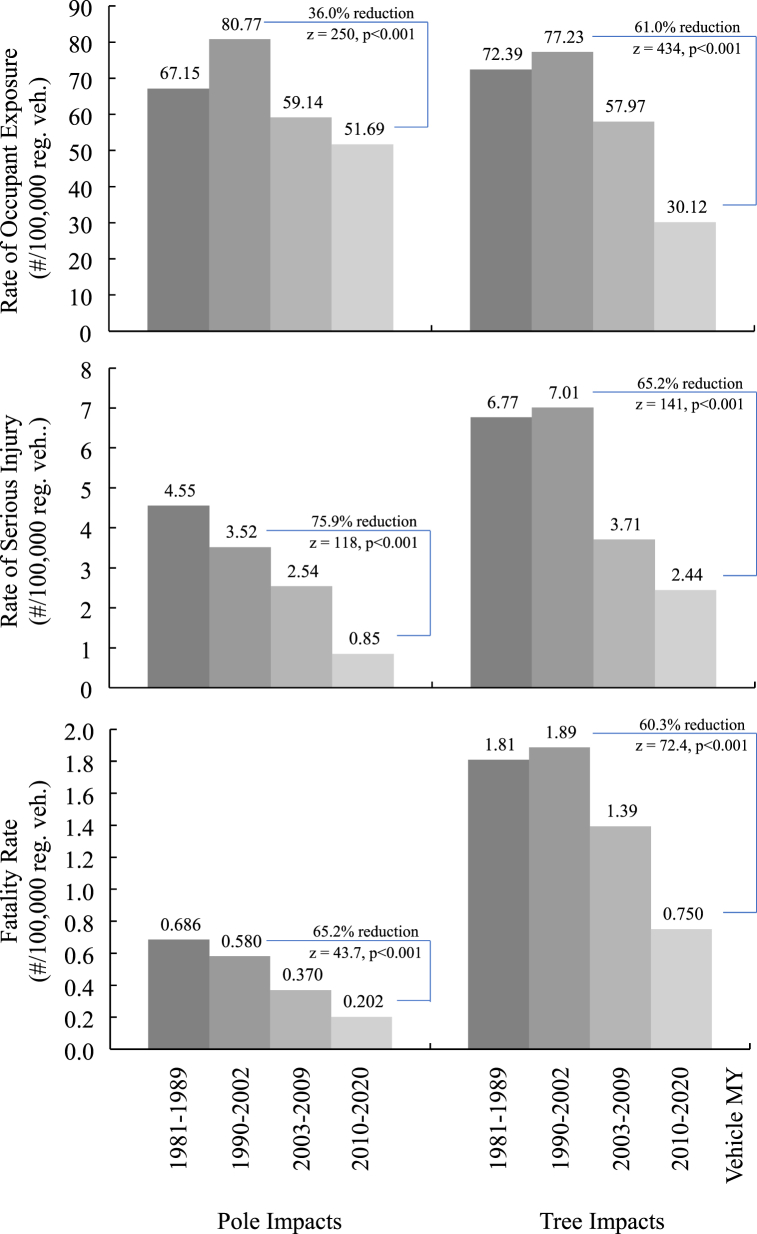
(top) rate of crash exposure to pole and tree impacts, (middle) rate of serious injury crashes into poles and trees and (bottom) rate of fatality from impacts of poles and trees (top-middle: 1990–2015 NASS-CDS, 2017–2020 CISS, 1990–2015 and 2017–2020 IHS Markit data, bottom: rate 1990–2020 FARS and 1990–2020 IHS Markit data).

Fig. 1 (top) shows the rate of occupants in towaway crashes into poles and tree impacts for all impact locations. The exposure rate decreased significantly in 2010–2020 MY vehicles with ESC compared to earlier models. The exposure rate was 80.77/100,000 registered vehicles with pole impacts in 1990–2002 MY vehicles compared to 51.69/100,000 registered vehicles in 2010–2020 MY vehicles. There was a 36.0 % 36.0 % (95 % CI, 35.8–36.3 %), z = 252, p < 0.001 reduction in pole impacts. The exposure rate was 77.23/100,000 registered vehicle into a tree in 1990–2002 MY vehicles compared to 30.12/100,000 registered vehicles in 2010–2020 MY vehicles. There was a 61.0 % (95 % CI, 60.8–61.2 %), z = 434, p < 0.001 reduction in tree impacts.

For all crash locations, the serious-injury rate was 3.52/100,000 registered vehicles in pole impacts in 1990–2002 MY vehicles compared to 0.85/100,000 registered vehicles in 2010–2020 MY vehicles. Fig. 1 (middle) shows there was a 75.9 % (95 % CI, 75.0–76.9 %), z = 116, p < 0.001 reduction in pole impacts with serious injury. The rate was 7.01/100,000 registered vehicle serious-injury impacts into a tree in 1990–2002 MY vehicles compared to 2.44/100,000 registered vehicles in 2010–2020 MY vehicles. There was a 65.2 % (95 % CI, 64.4–65.9 %), z = 141, p < 0.001 reduction in tree impacts with serious injury. Rear impacts into poles and trees associated with serious injury were essentially eliminated 2010–2020 MY vehicle crashes.

Table 5 shows the fatality rate in pole and tree impacts by location on the struck vehicle. The fatality rate dropped in newer vehicles equipped with ESC. The drops are statistically significant in a comparison of 1990–2003 MY vehicles to the 2010–2020 MY vehicle crash rates. For all crash locations, the fatality rate was 0.580/100,000 registered vehicles in pole impacts in 1990–2002 MY vehicles compared to 0.202/100,000 registered vehicles in 2010–2020 MY vehicles. Fig. 1 (bottom) shows the reduction in pole impacts was 65.2 % (95 % CI, 63.0–67.4 %), z = 43.7, p < 0.001. For all crash locations, the fatality rate was 1.89/100,000 registered vehicles into a tree in 1990–2002 MY vehicles compared to 0.750/100,000 registered vehicles in 2010–2020 MY vehicles. There was a 60.3 % (95 % CI, 59.0–61.5 %), z = 72.4, p < 0.001 reduction in tree impacts with 2010–2020 MY vehicles.

**Table 5:** Rate of Fatal Pole and Tree Impacts by location on the struck vehicle from 1990 to 2020 FARS and 1990–2020 IHS Markit.

**1990–2020 FARS**	**Fatality Rate per 100,000 Registered Vehicles**						**ESC**
**1990**–**2020 IHS Markit**	**Impact Direction**						
	**Front**	**Side**	**Rear**	**Rollover**	**Oth/Unk**	**Total**	
**Model years**	Pole Impact Fatalities						
1981–1989	0.334	0.201	0.0162	0.103	0.032	0.686	no
1990–2002	0.240	0.169	0.0157	0.113	0.043	0.580	no
2003–2009	0.153	0.0774	0.00728	0.084	0.048	0.370	mix
2010–2020	0.113	0.019	0.00269	0.037	0.030	0.202	yes
Total	0.215	0.130	0.0118	0.091	0.039	0.487	
**Model years**	Tree Impact Fatalities						
1981–1989	1.030	0.411	0.039	0.268	0.063	1.812	no
1990–2002	0.965	0.434	0.049	0.335	0.104	1.888	no
2003–2009	0.695	0.268	0.036	0.260	0.136	1.394	mix
2010–2020	0.464	0.0739	0.012	0.120	0.080	0.750	yes
Total	0.822	0.333	0.038	0.267	0.096	1.557	

There was a dramatic reduction in pole and tree impacts to the rear, side and front and in rollovers of the vehicle. Fatalities in rear impacts with poles and trees were significantly lower in 2010–2020 MY vehicles equipped with ESC than earlier models from 1990 to 2003 MY. For rear impacts, there was an 82.9 % (95 % CI, 71.3–94.4 %), z = 9.37, p < 0.001 reduction in fatality rate in pole impacts and 74.8 % (95 % CI, 67.8–81.9 %), z = 14.8, p < 0.001 reduction in tree impacts. For side impacts, there was an 88.6 % (95 % CI, 85.3–91.1 %), z = 33.1, p < 0.001 reduction in the fatality rate in pole impacts and 83.0 % (95 % CI, 80.8–85.2 %), z = 49.3, p < 0.001 reduction in tree impacts. For rollovers, there was an 66.9 % (95 % CI, 61.9–71.9 %), z = 19.8, p < 0.001 reduction in fatality rate in pole impacts and 64.3 % (95 % CI, 61.3–67.3 %), z = 32.7, p < 0.001 reduction in tree impacts. For front impacts, there was a 53.1 % (95 % CI, 49.3–56.9 %), z = 22.5, p < 0.001 reduction in fatality rate in pole impacts and 51.9 % (95 % CI, 50.0–53.8 %), z = 44.1, p < 0.001 reduction in tree impacts.

### 3.1 Individual NASS-CDS and CISS case review of serious injury in rear impacts with poles

There were 7 unweighted cases involving 8 seriously injured (MAIS 3+) occupants in rear impacts into a pole in NASS-CDS and CISS electronic cases. Table A1 summarizes the cases. None of the vehicles was equipped with ESC. All cases involved loss of control with vehicle yaw and unfavorable environmental conditions, including poor lighting, wet roadway. Six of the 7 cases were with multiple impacts. The posted speed varied from 56 to 80 km/h. One case involved a police vehicle involved in a pursuit. Another involved a vehicle separated into two parts with two occupants ejecting.

Fig. A1 shows photos of the rear of vehicle and occupant seat. The impacts were severe. Rear crush was determined in 5 cases and varied from 17 to 174 cm. Two types of damage were observed: one near the centerline of the vehicle and another offset to the side. Six cases involved severe (46+ cm) intrusion of the occupant compartment. The intrusion was severe enough to support the front seatback from rearward rotation. The case with police vehicle (#2007-8-8) involved equipment in the 2nd row supporting the driver seatback. The left rear seatback was folded down in case #2018-12-10, engaging with the driver seat on the outboard side. The occupant was morbidly obese increasing the load on the seatback in the rear impact.

### 3.2 Individual NASS-CDS and CISS case review of serious injury in rear impacts with trees

There were 14 unweighted cases involving 17 seriously injured (MAIS 3+F) occupants in the struck vehicle with a tree impact to the rear of the vehicle in NASS-CDS and CISS electronic cases. Table A2 summarizes the cases. None of the vehicles was equipped with ESC. Eleven of the 14 cases occurred in the dark. Twelve crashes involved more than one impact in the collision sequence. The posted speed varied from 56 to 113 km/h. One case involved a police vehicle with a fire. Fig. A2 shows photos of the rear of vehicle and occupant seat. The impacts were severe. Crush was determined in 11 of the 14 cases and varied from 15 to 252 cm.

### 3.3 Case summary of fatal impacts with poles and trees in 2010–2020 FARS

Summary information on fatal pole and tree impacts in the 2010–2020 FARS was tabulated and reviewed for vehicles equipped with ESC. Since the fractions were similar for pole and tree impacts, the average frequency is reported. The driver was male in 63.6 % of the crashes. The police reported alcohol use by 36.5 % of drivers. The police report 34.5 % of the crash were speed related and 29.6 % involved an adverse driving situation. Weather was a factor in 42.6 %. Seatbelts were not used in 39.8 % of the deaths. 16.3 % of occupants were completely or partially ejected.

## 4 Discussion

Electronic Stability Control (ESC) monitors the direction of the driver's steering input and the direction of the vehicle's heading. If the difference is above a threshold, braking is applied to selected wheels and engine torque is changed to bring the vehicle heading closer to the steering direction. ESC is helping drivers maintain vehicle heading and has significantly reduces off-road impacts into poles and trees. The driver's efforts to maintain control of the vehicle are enhanced with ESC, which has reduced the rate of serious injury and fatal crashes into poles and trees, if the driver actively steers the vehicle. The technology helps drivers avoid the very serious risk of off-road impacts into fixed objects by increasing directional control and reducing skids. Langwieder [53] noted that 25%–30 % of crashes involved some type of pre-impact skidding.

ESC requires the driver to steer the vehicle away from roadside hazards during emergency maneuvers. Drivers with reduced functions from alcohol and drug use or vision impairment or with adverse weather and road conditions may not make appropriate or timely steering inputs to maintain vehicle control. In these situations, they may not benefit from ESC. The 2010–2020 FARS cases indicate alcohol involvement and adverse weather in pole and tree impacts with vehicles equipped with ESC, which is consistent with factors observed in a review of literature on single-vehicle crashes [54]. This may explain the continued occurrence of off-road impacts with trees and poles, but at a substantially lower rate than in 1990–2003 MY vehicles. There has to be active steering for ESC to function and assist in providing a heading away from fixed objects.

The safety benefits of ESC are remarkable and somewhat fortuitous. In the early development of ESC at GM Research Laboratories and Delphi, the projected benefits were quite modest because of essentially no experience with ESC in real-world conditions where the technology has proven effective in the field. Efforts to conduct aggressive handling tests of the type seen in fatal off-road crashes were prohibited at the GM Proving Grounds for reasons of test-driver safety [55,56]. The positive effects of ESC were born-out in the field accident data, not proving ground tests of vehicles equipped with ESC.

Riexinger et al. [57] evaluated ESC effects in reducing rollover crashes. Loss of control occurred in 29.7 % of 1,339,407 vehicle rollovers in 2006–2015 NASS-CDS. Only 177,644 of vehicles were equipped with ESC. They found that ESC reduced rollovers due to loss of control by 50.6 %. ESC effectiveness was estimated to reduce all rollovers by 13.3 %. In this study, there was a 66.9 % reduction in fatal rollovers into poles and 64.3 % reduction into trees. The reductions are higher than the Riexinger et al. [57] estimates for the specific type of fixed object impacts investigated here. This study found significant reductions for all types of pole and tree impacts in vehicles equipped with ESC.

### 4.1 Rear-leading crashes

Tractor-trailer impacts to the rear of a vehicle have the highest risk (2.71 %) of serious injury in the struck vehicle (56). The second-highest risk (1.49 %) was a rear impact with a fixed object, such as a tree, pole or embankment. Rear impacts with fixed objects involve off-road excursion with the vehicle rear-leading into the object. The risk for serious injury was higher than tractor-trailer impacts at 3.57 % for tree or bush impacts and 2.90 % for pole impacts.

**Fig. 2:**
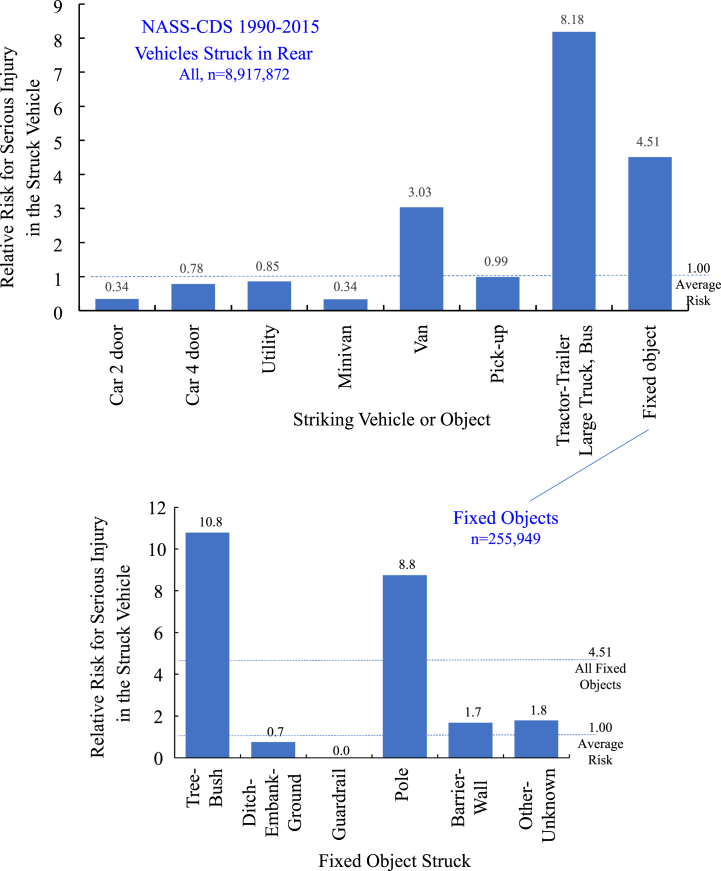
(top) relative risk for serious injury to an occupant of a vehicle struck in the rear by the impacting vehicle or object and (bottom) relative risk by fixed object impacts to the rear of a struck vehicle from 1990 to 2015 NASS-CDS, data from Viano, Parenteau (2022).

Fig. 2 (top) shows the relative risk for serious injury to an occupant of a vehicle struck in the rear by the type of impacting vehicle or object (56). The relative risk was 8.18 times for rear impacts by a tractor-trailer, large truck or bus and 4.51 times for rear impacts with a fixed object. The underlying risk was highest in tractor-trailer impacts (2.71 %), followed by a fixed-object impacts (1.49 %). The average risk was 0.33 %.

Fig. 2 (bottom) shows the relative risk for serious injury in fixed object impacts to the rear of the struck vehicle (56). The highest relative risk in fixed-object impacts were in tree-brush (10.8 times) and pole (8.8 times) impacts. Electronic cases of rear impacts into trees and poles were downloaded and reviewed. A surprising finding was only older vehicles were involved in pole or tree rear impacts. None of the electronic cases involved a vehicle equipped with Electronic Stability Control (ESC). This finding prompted the current study of pole and tree impacts in NASS-CDS, CISS and FARS.

The review of rear impacts with poles and trees in NASS-CDS and CISS is summarized in the Appendix. None of the vehicles was equipped with ESC. The collision damage seen in Figs. A1 and A2 in vehicles not equipped with ESC involves high forces per unit width with pole and tree impacts and very high speeds of impact. The collision forces cause intrusion with the narrow aspect ratio of poles and trees that concentrate forces on a narrow aspect of the vehicle structures. The concentrated forces make improvements in vehicle structures difficult. It is better to avoid fixed object impacts than to manage the forces by crashworthiness systems. The reduction of this class of off-road impact is extremely beneficial for occupant protection and is positive for crash avoidance technologies like ESC. Figs. A1 and A2 show significant collision forces and intrusion. These types of crashes are greatly reduced, almost eliminated, in vehicles equipped with ESC. The significantly lower off-road impacts with poles and trees indicate drivers are actively steering the vehicle in the collision sequence. ESC changes the vehicle heading and limits the excursions off-road.

### 4.2 Limitations

No effort was made to consider ways that ESC might be improved. The capabilities of the technology are already remarkable, but there may be ways to enhance its performance. There are other limitations that exist with all field data that confound the evaluation of crash avoidance features (ESC, automatic emergency braking) and crash protection features (depowered airbags, side curtains). The age of the vehicle, socioeconomic status of the driver, alcohol and drug use, age and size of the occupants are factors that influence crash injury and death. There was no attempt to include these factors in the analysis or make adjustments for them in the data. The type of evaluation conducted here is typical of the methods used by NHTSA and other researchers for the evaluation of safety technologies. Kahane [58] provides an overview of the important factors influencing field accidents and injuries. The NASS-CDS analysis combined number of years of field data from 1990 to 2015. Various AIS coding versions were used by the NHTSA crash investigators, including AIS98, based on AIS95, AIS08, based on AIS05, and AIS15. The effect of AIS coding variations was not analyzed in this study.

## 5 Conclusions

ESC helps the driver maintain vehicle heading. It significantly reduced the rate of serious injury and fatality in off-road impacts with poles and trees. The benefits of ESC may not be realized with impairments when the driver does not appropriately steer the vehicle.

## Data availability

The field data is publicly available through the US Department of Transportation, Highway Traffic Safety Administration. The FARS data can be accessed at: https://www.nhtsa.gov/research-data/fatality-analysis-reporting-system-fars#:∼:text=FARS%20is%20a%20nationwide%20census,in%20motor%20vehicle%20traffic%20crashes. The CISS data can be accessed at: https://www.nhtsa.gov/crash-data-systems/crash-investigation-sampling-system. The NASS-CDS data can be accessed at: https://www.nhtsa.gov/crash-data-systems/national-automotive-sampling-system. There are online viewers for each case and the data can be analyzed using statistical analysis software, such as SAS. Vehicle registration data for light vehicles can be accessed at IHS Markit: https://ihsmarkit.com/products/auto-market-statistics-vio-vin.html.

## Ethics statement

The authors have followed all ethical requirements for publishing a research study.

## CRediT authorship contribution statement

**David C. Viano:** Writing – review & editing, Writing – original draft, Methodology, Conceptualization. **Chantal S. Parenteau:** Formal analysis. **Eric R. Teoh:** Methodology, Formal analysis, Data curation.

## Declaration of competing interest

The authors declare the following financial interests/personal relationships which may be considered as potential competing interests: The authors report financial support for the article publishing charges were provided by Institute for Injury Research. David C Viano reports a relationship with Institute for Injury Research that includes: board membership. The other authors declare no known competing financial interests or personal relationships that could have appeared to influence the work reported in this paper.

## References

[1] Tingvall C., Krafft M., Kullgren A., Lie A. (2003).

[2] Aga M., Okada A. (2003).

[3] Lie A., Tingvall C., Krafft M., Kullgren A. (2004). The effectiveness of ESP (Electronic Stability Program) in reducing real life accidents. Traffic Inj. Prev..

[4] Farmer C.M. (2004). Effect of electronic stability control on automobile crash risk. Traffic Inj. Prev..

[5] Dang J.N. (2004). Preliminary results analyzing the effectiveness of electronic stability control (ESC) systems. DOT-HS.

[6] Farmer C.M. (2006). Effects of electronic stability control: an update. Traffic Inj. Prev..

[7] Erke A. (2008). Effects of electronic stability control (ESC) on accidents a review of empirical evidence. Accid Anal Prev. Jan.

[8] Ferguson S.A. (2007). The effectiveness of electronic stability control in reducing real-world crashes: a literature review. Traffic Inj Prev. Dec.

[9] Høye A. (2011). The effects of electronic stability control (ESC) on crashes--an update. Accid Anal Prev. May.

[10] Af Wahlberg A.E., Dorn L. (2024). The effects of Electronic Stability Control (ESC) on fatal crash rates in the United States. J. Saf. Res..

[11] Bahouth G. (2006). SAE 2006-01-0925.

[12] Chouinard A., Lécuyer J.F. (2011). A study of the effectiveness of electronic stability control in Canada. Accid Anal Prev. Jan.

[13] Dang J. (2007). Statistical analysis of the effectiveness of electronic stability control (ESC) systems. DOT HS.

[14] Green P.E., Woodrooffe J. (2006). The estimated reduction in the odds of loss-of-control type crashes for sport utility vehicles equipped with electronic stability control. J. Saf. Res..

[15] Kallan M.J., Jermakian J.S. (2008). 52^nd^ AAAM Conference.

[16] Kim T., Bose D., Foster J., Bollapragada V., Crandall J.R., Clauser M., Kerrigan J.R. (2017). Identification of characteristics and frequent scenarios of single-vehicle rollover crashes during pre-ballistic phase; part 1 -A descriptive study. Accid Anal Prev. Oct.

[17] Lie A., Tingvall C., Krafft M., Kullgren A. (2005).

[18] Lyckegaard A., Hels T., Bernhoft I.M. (2015). Effectiveness of electronic stability control on single-vehicle accidents. Traffic Inj. Prev..

[19] MacLennan P.A., Marshall T., Griffin R., Purcell M., McGwin G., Rue L.W. (2008). Vehicle rollover risk and electronic stability control systems. Inj Prev. Jun.

[20] Scully J., Newstead S. (2008). Evaluation of electronic stability control effectiveness in Australasia. Accid Anal Prev. Nov.

[21] Sivinski R. (2011).

[22] Thomas P., Frampton R. (2007). Paper 07-0184, ESV Conference.

[23] Weekes A., Avery M., Frampton R., Thomas P. (2009).

[24] Riexinger L.E., Gabler H.C. (2020). Expansion of NASS/CDS for characterizing run-off-road crashes. Traffic Inj Prev. Oct.

[25] Keall M.D., Newstead S. (2021). Evaluation of the effectiveness of vehicle roll stability control (RSC) for high center of gravity light passenger vehicles in Australasia. Traffic Inj. Prev..

[26] Padmanaban J., Shields L.E., Scheibe R.R., Eyges V.E. (2008). A comprehensive review of rollover accidents involving vehicles equipped with Electronic Stability Control (ESC) systems. Ann Adv Automot Med. Oct.

[27] Strandroth J., Rizzi M., Olai M., Lie A., Tingvall C. (2012). The effects of studded tires on fatal crashes with passenger cars and the benefits of electronic stability control (ESC) in Swedish winter driving. Accid Anal Prev. Mar.

[28] Sternlund S. (2019). The Effectiveness of centerline rumble strips (CLRS) on two-lane carriageways in Sweden on injury accident risk for cars equipped with electronic stability control (ESC) and cars without ESC. Traffic Inj. Prev..

[29] Fildes B., Keall M., Thomas P., Parkkari K., Pennisi L., Tingvall C. (2013). Evaluation of the benefits of vehicle safety technology: the MUNDS study. Accid Anal Prev. Jun.

[30] Griffin R., McGwin G., Kerby J. (2018). Decomposition analysis of the effects of vehicle safety technologies on the motor vehicle collision-related mortality rate from 1994 to 2015. Traffic Inj. Prev..

[31] Page Y., Cuny S., Zangmeister T., Kreiss J.P., Hermitte T. (2009). The evaluation of the safety benefits of combined passive and on-board active safety applications. Ann Adv Automot Med.

[32] Markkula G., Benderius O., Wolff K., Wahde M. (2013). Effects of experience and electronic stability control on low friction collision avoidance in a truck driving simulator. Accid Anal Prev. Jan.

[33] Papelis Y.E., Watson G.S., Brown T.L. (2010). An empirical study of the effectiveness of electronic stability control system in reducing loss of vehicle control. Accid. Anal. Prev..

[34] Vadeby A., Wiklund M., Forward S. (2011). Car drivers' perceptions of electronic stability control (ESC) systems. Accid Anal Prev. May.

[35] Rubin-Brown C.M., Jenkins R.W., Whitehead T., Burns P.C. (2009). Could ESC (Electronic Stability Control) change the way we drive?. Traffic Inj Prev. Aug.

[36] NHTSA (2007). Federal motor vehicle safety standards; electronic stability control systems. Correction. Federal Register.

[37] NHTSA (2007). FMVSS 126 electronic stability control systems. Final regulatory impact analysis, office of regulatory analysis and evaluation. National Center for Statistics and Analysis, NHTSA.

[38] Holdridge J.M., Shankar V.N., Ulfarsson G.F. (2004). The crash severity impacts of fixed roadside objects. J. Saf. Res..

[39] IIHS. 2022. https://www.iihs.org/topics/fatality-statistics/detail/collisions-with-fixed-objects-and-animals, posted (May).

[40] NHTSA (2022). Traffic safety facts 2020 data. DOT HS.

[41] Stewart T. (2022). DOT HS 813 266.

[42] Lund A.K. (2004). Frontal new car assessment program (NCAP) request for comments. Docket NHTSA-2004-18765, Insurance Institute for Highway Safety.

[43] Lockhart P.A., Cronin D.S., Watson B. (2012). Frontal impact response for Pole crash scenarios. Traffic Inj. Prev..

[44] IIHS (2021).

[45] Pintar F.A., Maiman D.J., Yoganandan N. (2007). Injury patterns in side pole crashes annu proc assoc. Adv Automot Med.

[46] Digges K.H., Eigen A.M. (2005). IRCOBI Conference.

[47] Viano D.C., Parenteau C.S., Edwards M.L. (2007). Rollover injury: effects of near- and far-seating position, belt use and number of quarter rolls. Traffic Inj. Prev..

[48] NHTSA (2014). FARS–Fatality analysis reporting system. DOT HS.

[49] NHTSA (2008). National automotive sampling system. DOT HS.

[50] Zhang F., Subramanian R., Chen C.L., Young Noh E.Y. (2019). Crash investigation sampling system: design overview, analytic guidance, and FAQs. DOT HS.

[51] Montgomery D.C., Runger G.C. (2014).

[52] Viano D.C., Parenteau C.S. (2022). Significance of tractor-trailer impacts to the rear of light vehicles. Traffic Inj. Prev..

[53] Langwieder K. (1999). Characteristics of car accidents in the pre-crash phase. JSAE Spring Conference Proceedings.

[54] Bucsuhazy K., Zuvala R., Valentova V., Ambros J. (2022). Factors related to severe single-vehicle tree crashes: in-depth crash study. PLoS One.

[55] Viano D.C., Ridella S.A. (1996).

[56] Viano D.C., Parenteau C.S. (2003). SAE 2003-01-0169.

[57] Riexinger L., Sherony R., Gabler H. (2019).

[58] Kahane C.J. (2015). Lives saved by vehicle safety technologies and associated Federal Motor Vehicle Safety Standards, 1960 to 2012 – passenger cars and LTVs – with reviews of 26 FMVSS and the effectiveness of their associated safety technologies in reducing fatalities, injuries, and crashes. DOT HS 812 069, National Highway Traffic Safety Administration.

