# Supplementary material for: Effect of ESC (electronic stability control) on tree and pole impacts with focus on rear impacts
Source: Heliyon. 2024 Sep 7;10(18):e37591. doi: 10.1016/j.heliyon.2024.e37591 (PMC11438009; doi:10.1016/j.heliyon.2024.e37591)
Supplement: Multimedia component 1 [file mmc1.pdf]

Appendix

Table A1: NASS-CDS/CISS cases with a seriously injured occupant involved in a rear pole impact.

#	RAT WGT	YEAR	PSU	CASE NO	Posted speed (mph)	MY	VEH	Delta V (km/h)	YAW	# imp	Max crush (cm)	FACTORS	OCC	AGE (yr)	Restr.	MAIS	Injury
1	8.6	2005	79	17	35	2000	Chevy Camaro	Severe	CCW	2	Unk	Dark, rain, wet, intrusic	D	18	L-S	3	Humerus fx
2	8.9	2006	74	117	50	2000	Saturn LS1	33	CW	2	58	Rain, wet, intrusion	D	18	None	5 F	Cerebrum
3	3.6	2006	82	36	40	1991	Saturn LS1	88	CW	2	145	Intrusion, alcohol, darl	RF	22	None	4	Cerebrum
4	223.2	2007	8	8	40	2004	Chevrolet Impala*	Severe	CW	4	174	Dark, rain, wet, drugs	D	40	None	4	Cerebrum
5	24.0	2007	73	8	40	2004	Pontiac Grand Prix	Severe	CCW	6	17	Dark, alcohol (0.26)	D	34	None	3	Thoracic spine
6	56.8	2011	78	134	50	2007	Honda Accord**	Severe	CCW	5	Unk	Dark, ejection, vehicle	LR	18	None	6 F	Cervical spine
	"--"	"--"	"--"	"--"	"--"	"--"	"--"	"--"	"--"	"--"		split	RR	17	None	3	Vault fx
7	459.3	2018	12	10	45	2003	Toyota Matrix	75	CCW	1	111	Speeding, wet, curve	D	40	L-S	5	Thoracic spine

D: Driver, RF: Right-front, LR: Left rear, RR: Right-rear, SB: Seatback, F: Fatality, CW: Clockwise, CCW: Counter-clockwise
PSU: primary sampling unit, see NASS-CDS User Manual, DOT HS 812 066, Delta V: change in vehicle velocity, L-S: lap-shoulder belted
Rat wgt: Ration inflation factor, see NASS-CDS User Manual, fx: fracture
* Police vehicle
**Only six-cylinder Accords had ESC for 2006-2007.

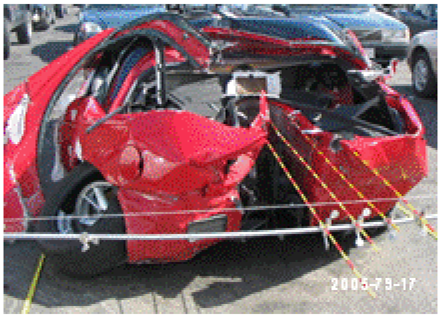

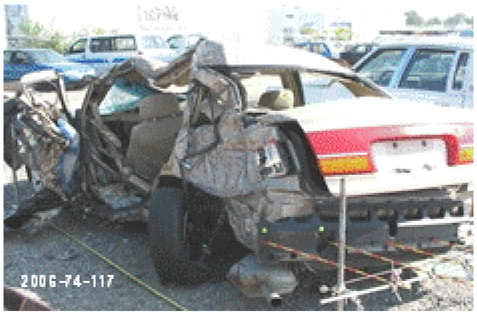

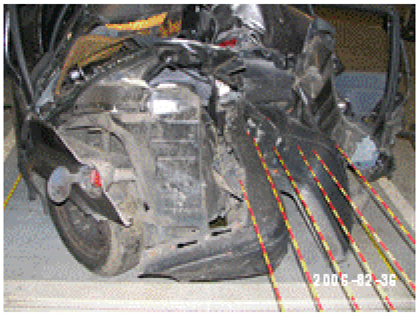

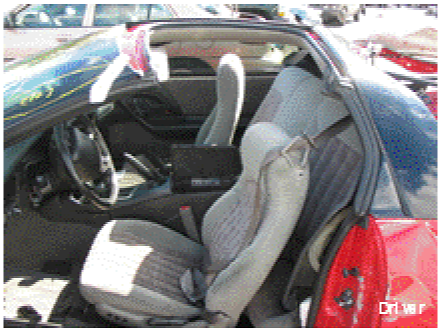

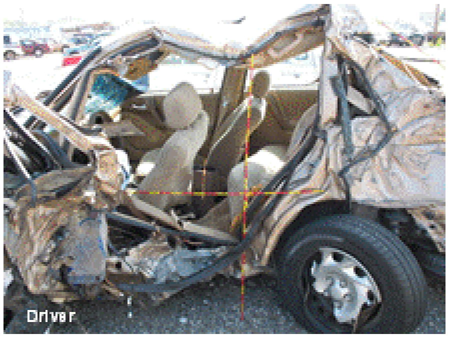

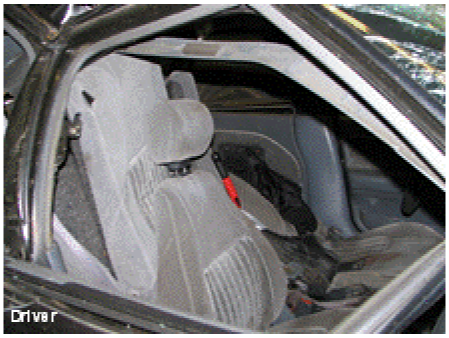

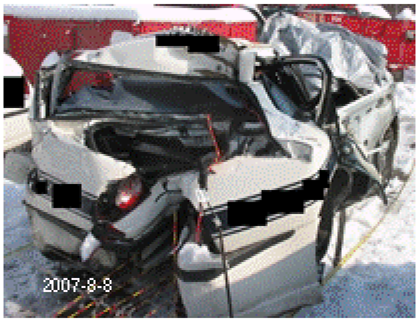

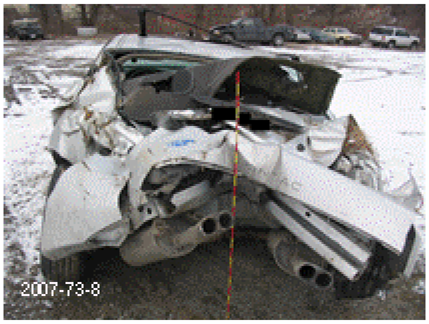

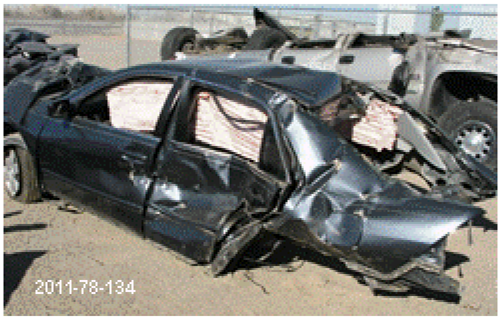

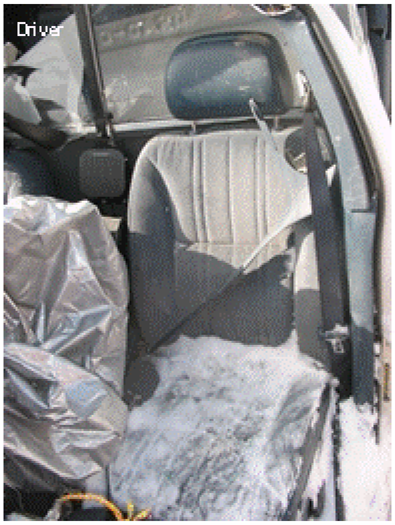

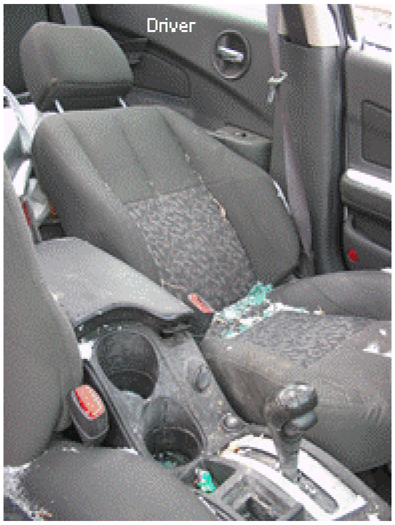

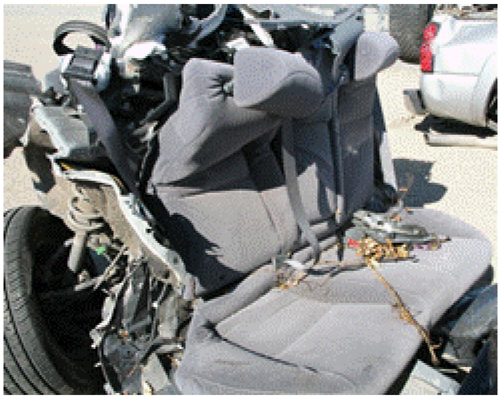

LR & RR
(ejection)

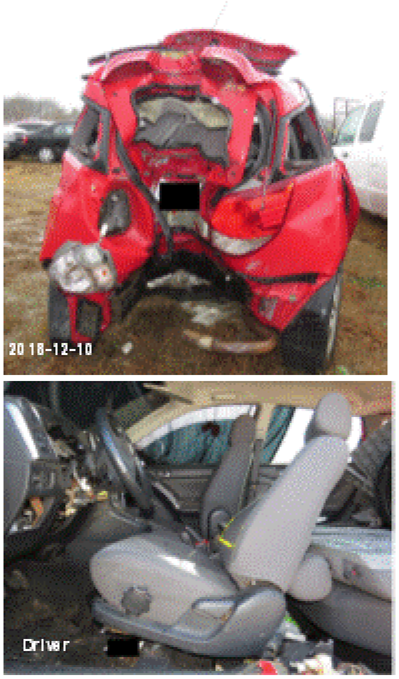

Figure A1: Case summary with seriously injured occupants in rear impacts with poles.

Table A2: NASS-CDS/CISS cases with a seriously injured occupant involved in a rear tree impact.

#	RAT WGT	YR	PSU	CASE NO	Posted speed (mph)	MY	VEH	Delta V (km/h)	Yaw	Max crush (cm)	# imp	FACTORS	OCC	AGE	Restr.	MAIS	Injury
1	171.2	2004	48	114	40	1992	Ford Crown Victoria	Severe	CW	181	2	Dark, curve, speeding,	D	40	None	5	DAI
2	25.6	2005	12	116	55	1995	Chevrolet Lumina	26	CCW	38	2	Dark	D	17	None	4 F	Cerebrum lac
3	30.7	2006	75	61	45	1996	Honda Civic		CW	80	3	Dark, curve, speeding,	LR	17	L-S	3	Femur
4	72.7	2007	47	78	70	1997	Chevrolet Camaro	65	CCW	112	3	Dark, maneuver	CR	13	None	F	
	"--"	"--"	"--"	"--"	"--"	"--"	"--"	"--"	"--"	"--"	"--"	"--"	RR	15	None	F	
5	18.4	2008	41	252	45	2005	Ford Crown		CCW	Unk	2	Dark but lighted,	D	31		5 F	Whole body
6	38.6	2009	45	108	40	2000	Chevrolet Impala	Severe	CCW	23	1	Dark, curve, rain, wet	D	33	L-S	3	Lung
7	122.3	2009	76	73	55	2004	Ford Expedition	46	CW	66	3	Alcohol, curve, daylight	D	30	None	3	Radius fx
8	57.1	2010	43	263	45	2004	Dodge Durango		CCW	23	4	Curve, sleet/hail, slushy	CR	52	None	5	Thoracic spine
9	11.3	2010	81	1	35	2004	Chevrolet Impala		CW	252	1	Alcohol, curve, intrusion,	D	18	L-S	6 F	Brainstem
	"--"	"--"	"--"	"--"	"--"	"--"	"--"	"--"	"--"	"--"	"--"	dark but lighted	LR	14	L-S	5	Pelvis fx
	"--"	"--"	"--"	"--"	"--"	"--"	"--"	"--"	"--"	"--"	"--"	"--"	RR	14	L-S	3	Femur fx
10	33.4	2012	79	16	50	1998	MB E-class	61	CCW	174	3	Speeding, dark but	D	25	L-S	F	NA
11	2.9	2013	82	110	40	2002	Ford Focus	32	CW	Unk	4	Dark but lighted,	D	21	L-S	F	NA
12	91.8	2014	13	27		1994	Chevrolet Camaro	44	CCW	96	9	Speeding, dark but	RF	28	Unk	F	NA
13	175.7	2015	48	108	50	2007	2007 Toyota Scion	19	CCW	15	2	Alcohol, curve, daylight	D	19	None	3 F	Thoracic injury
14	47.4	2015	12	61	55	1987	Chevrolet Camaro	87	CW	139	2	Negotiating a curve,	D	25	None	F	NA
	"--"	"--"	"--"	"--"	"--"	"--"	"--"	"--"	"--"	"--"	"--"	speeding, dark	RF	31	None	F	NA

D: Driver, RF: Right-front, LR: Left rear, CR: Center-rear, RR: Right-rear, CW: Clockwise, CCW: Counter-clockwise, L-S: lap-shouler belt
PSU: primary sampling unit, see NASS-CDS User Manual, DOT HS 812 066, Delta V: change in vehicle velocity, L-S: lap-shoulder belted
Rat wgt: Ration inflation factor, see NASS-CDS User Manual, fx: fracture, SB: Seatback, F: Fatality, Lac: laceration, DAI: diffuse axonal injury
* Police vehicle
**2007 Scion does not have ESC

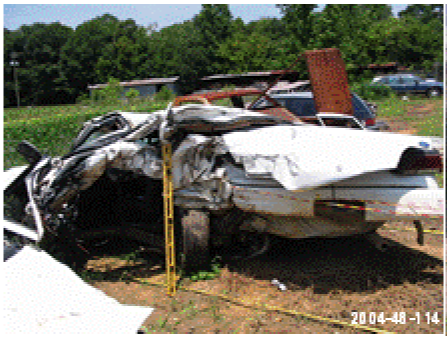
2004-48-114

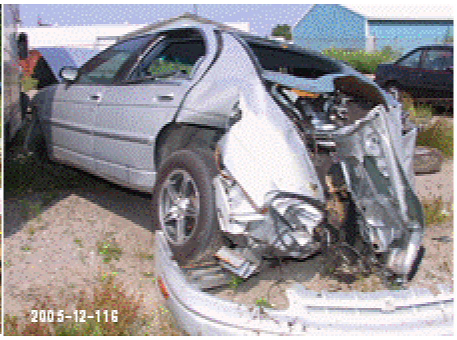
2005-12-116

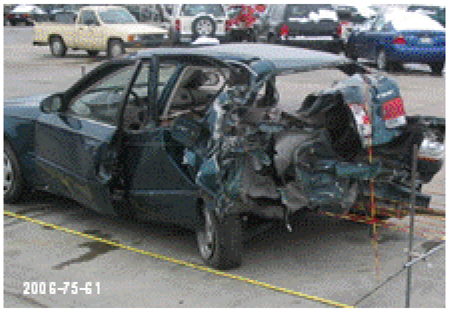
2006-75-61

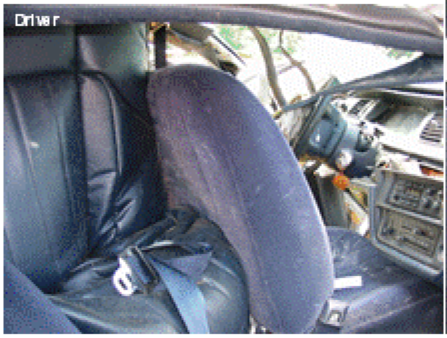
Driver

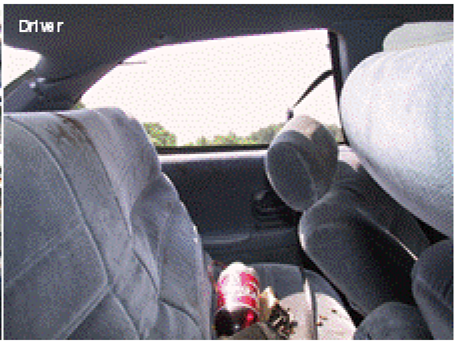
Driver

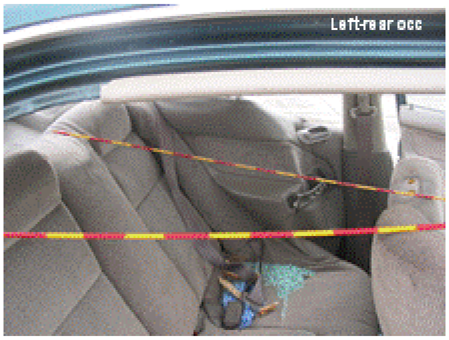
Left-rear occ

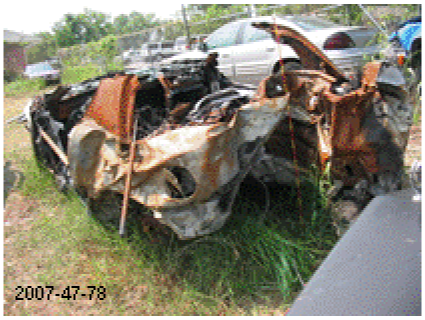
2007-47-78

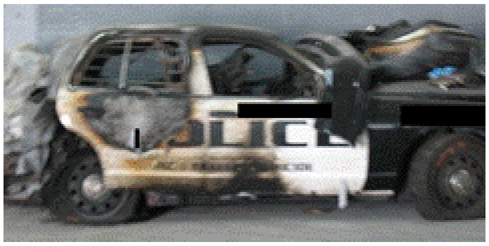

2008-41-252

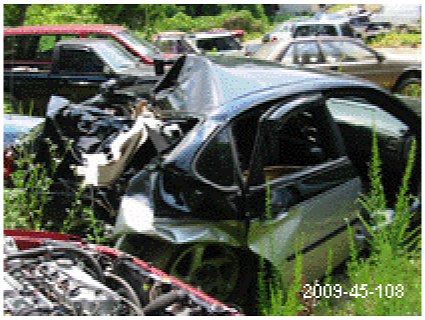
2009-45-108

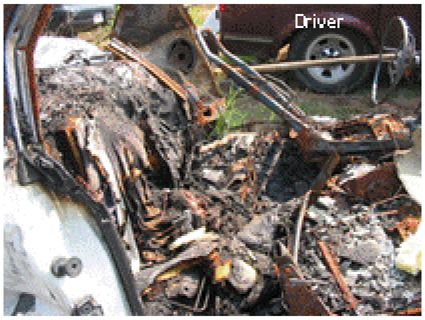
Driver

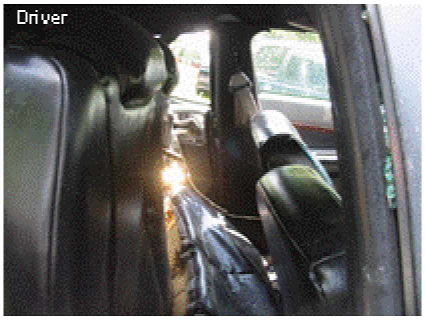
Driver

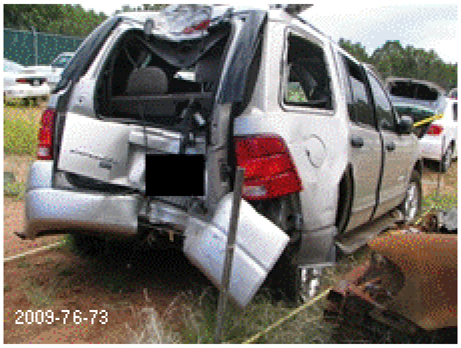
2009-76-73
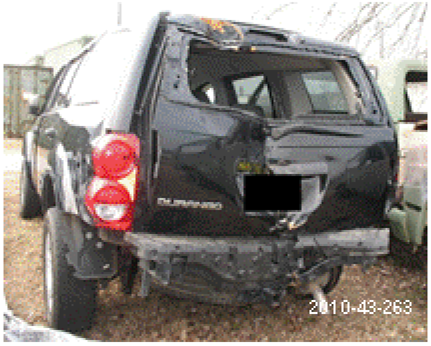
2010-43-263
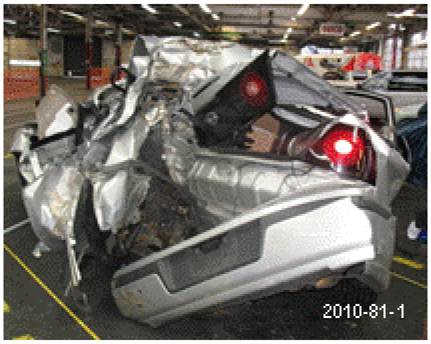
2010-81-1
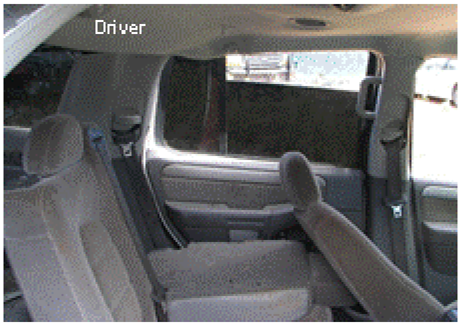
Driver
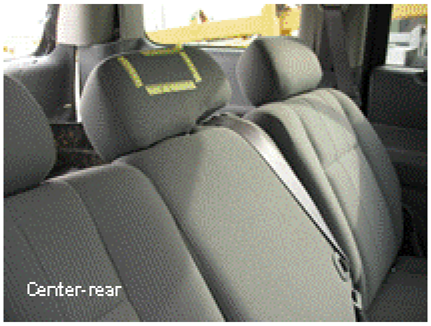
Center-rear
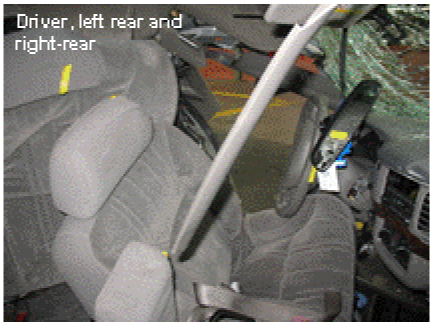
Driver, left rear and
right-rear
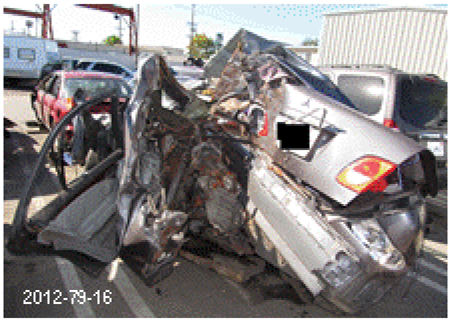
2012-79-16
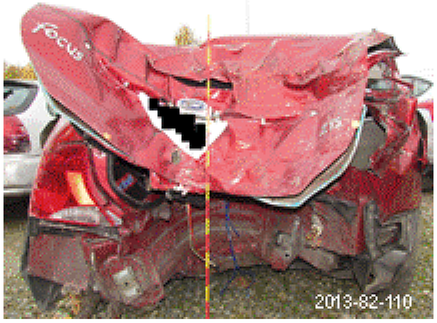
2013-82-110
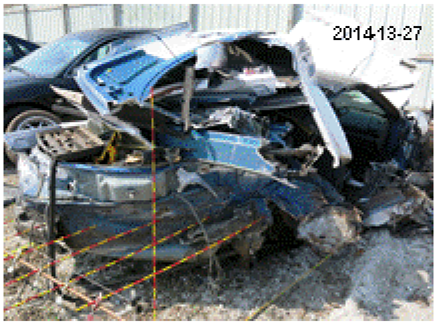
2014-13-27
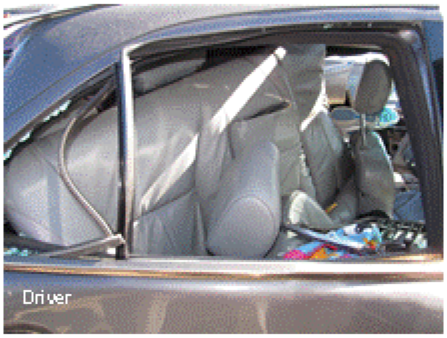
Driver
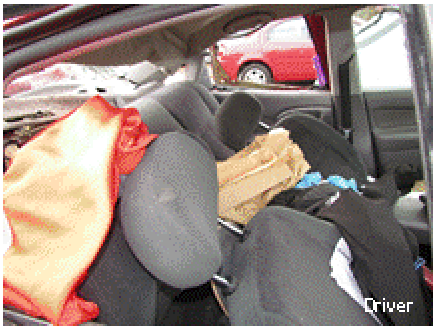
Driver
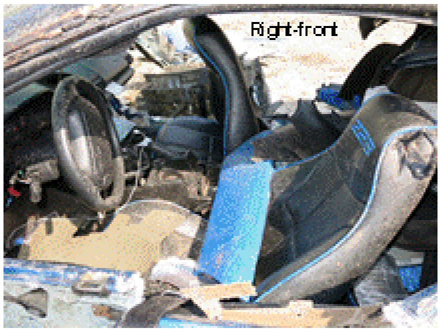
Right-front

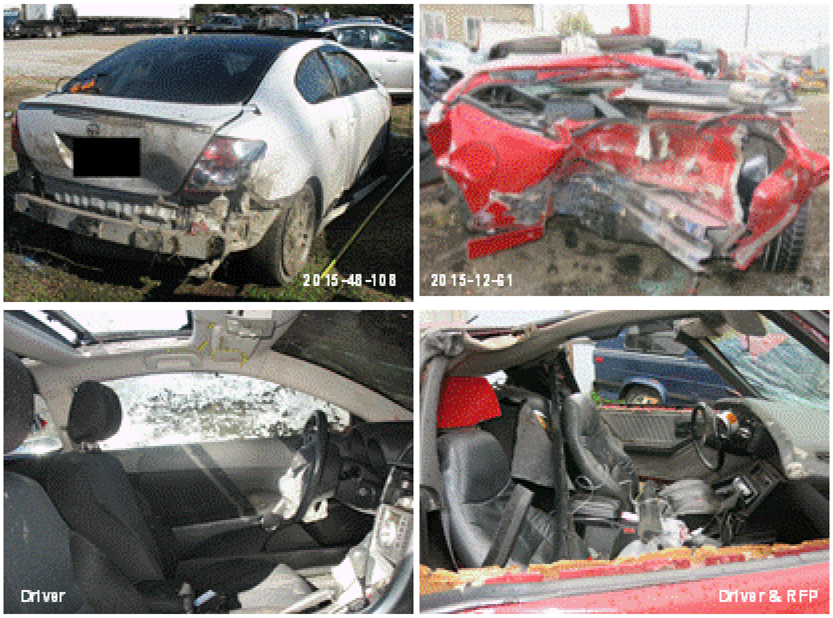

Figure A2: Case summary with seriously injured occupants in rear impacts with trees.
